# Outcomes following oesophagectomy in patients with oesophageal cancer: a secondary analysis of the ICNARC Case Mix Programme Database

**DOI:** 10.1186/cc7868

**Published:** 2009-06-01

**Authors:** Daniel P Park, Catherine A Welch, David A Harrison, Thomas R Palser, David A Cromwell, Fang Gao, Derek Alderson, Katherine M Rowan, Gavin D Perkins

**Affiliations:** 1 Division of Medical Sciences, University of Birmingham, Vincent Drive, Edgbaston, Birmingham, B15 2TT, UK.; 2 University of Warwick, Warwick Medical School Clinical Trials Unit, Gibbet Hill Road, Coventry, CV4 7AL, UK.; 3 Academic Department of Anaesthesia, Critical Care and Pain, Heart of England NHS Foundation Trust, Bordesley Green East, Birmingham, B9 5SS, UK.; 4 Intensive Care National Audit & Research Centre, Tavistock House, Tavistock Square, London, WC1H 9HR, UK.; 5 Clinical Effectiveness Unit, Royal College of Surgeons of England, 35-43 Lincoln's Inn Fields, London, WC2A 3PE, UK.; 6 University of Birmingham, College of Medical and Dental Sciences, School of Cancer Sciences, Academic Department of Surgery, Room 29, 4th Floor, Queen Elizabeth Hospital, Metchley Park Road, Edgbaston, Birmingham, B15 2TH, UK.

## Abstract

**Introduction:**

This report describes the case mix and outcomes of patients with oesophageal cancer admitted to adult critical care units following elective oesophageal surgery in England, Wales and Northern Ireland.

**Methods:**

Admissions to critical care following elective oesophageal surgery for malignancy were identified using data from the Intensive Care National Audit and Research Centre (ICNARC) Case Mix Programme Database. Information on admissions between December 1995 and September 2007 were extracted and the association between in-hospital mortality and patient characteristics on admission to critical care was assessed using multiple logistic regression analysis. The performance of three prognostic models (Simplified Acute Physiology Score (SAPS) II, Acute Physiology and Chronic Health Evaluation (APACHE) II and the ICNARC physiology score) was also evaluated.

**Results:**

Between 1995 and 2007, there were 7227 admissions to 181 critical care units following oesophageal surgery for malignancy. Overall mortality in critical care was 4.4% and in-hospital mortality was 11%, although both declined steadily over time. Eight hundred and seventy-three (12.2%) patients were readmitted to critical care, most commonly for respiratory complications (49%) and surgical complications (25%). Readmitted patients had a critical care unit mortality of 24.7% and in-hospital mortality of 33.9%. Overall in-hospital mortality was associated with patient age, and various physiological measurements on admission to critical care (partial pressure of arterial oxygen (PaO_2_):fraction of inspired oxygen (FiO_2_) ratio, lowest arterial pH, mechanical ventilation, serum albumin, urea and creatinine). The three prognostic models evaluated performed poorly in measures of discrimination, calibration and goodness of fit.

**Conclusions:**

Surgery for oesophageal malignancy continues to be associated with significant morbidity and mortality. Age and organ dysfunction in the early postoperative period are associated with an increased risk of death. Postoperative serum albumin is confirmed as an additional prognostic factor. More work is required to determine how this knowledge may improve clinical management.

## Introduction

Oesophagectomy is a major surgical procedure involving resection of all or part of the oesophagus and subsequent restoration of continuity to the gastrointestinal tract. The majority of oesophagectomies are performed electively as a curative treatment for oesophageal malignancy, now the eighth most commonly diagnosed malignancy worldwide [1].

The incidence of oesophageal cancer increases with age, and the disease is more common in men than women. Known risk factors include smoking, obesity and alcohol consumption. The prognosis for the condition continues to be poor, with many patients presenting too late or medically unfit for curative treatment. Approximately one in five patients with oesophageal cancer in England currently undergo surgical resection [2], and Hospital Episode Statistics show that approximately 1500 to 2000 oesophagectomies are performed in England annually [3].

Despite improvements in perioperative care, there continues to be considerable mortality and morbidity associated with oesophagectomy. In 2004, a systematic review of outcomes following oesophagectomy found an average mortality rate of 8.8% [4]. Postoperative complications are common, and include primary surgical events such as anastomotic or chylous leaks, as well as medical sequelae including pneumonia, acute lung injury and cardiac arrhythmias.

It is common practice to admit patients to the critical care unit following elective oesophageal surgery for malignancy, and the aim of this study is to describe the characteristics and outcomes of these patients. We also look at risk factors for a poor outcome following surgery. Much of the existing literature has concentrated on examining preoperative or intraoperative prognostic criteria [5-10]; however, we also identify risk factors in the early postoperative period that are associated with increased mortality. Finally the performance of three prognostic models commonly used in critical care is evaluated in this population.

## Materials and methods

### Case Mix Programme database

The Case Mix Programme (CMP) database is comprised of details of admissions to general adult critical care units across England, Wales and Northern Ireland. Participation in the voluntary programme has steadily increased over time, and currently data are submitted from more than 80% of such facilities. Intensive care units, high-dependency units and combined units are all represented. Data collection are carried out prospectively by trained staff according to strict protocol and exact definitions [11]. The data undergo both local and central validation for completeness, illogicalities and inconsistencies [12]. Data collection has been independently assessed to be of high quality [13], and the Intensive Care National Audit and Research Centre (ICNARC) has gained approval for the CMP database under Section 60 of the Health and Social Care Act 2001 (Approval Number PIAG 2-10(f)/2005).

### Oesophagectomy case definition and search strategy

The CMP database codes primary and secondary reasons for admission, according to the specifically designed ICNARC Coding Method [14]. Because operative procedures are not individually coded, we devised the following search strategy to identify eligible individuals. Patient admissions were included in the study if they had been coded as elective or scheduled surgical episodes, with a primary or secondary reason for admission to critical care of oesophageal or gastro-oesophageal junction malignancy, and if the source of admission was directly or indirectly from the operating theatre.

The database was searched for patients admitted to a critical care unit following elective oesophageal surgery for malignancy between 1 December 1995 and 30 September 2007. During this period data from 181 critical care units were available, with a total of 563,290 admissions for all indications.

### Data retrieval

#### Patient characteristics

Information was extracted on patient age and sex, and various physiological measurements taken on admission to the critical care unit. Three severity of illness scores were also calculated: the Acute Physiology and Chronic Health Evaluation (APACHE) II score [15], the ICNARC score [16] and the Simplified Acute Physiology Score (SAPS) II [17].

The APACHE II score comprises 12 physiological variables, with additional weighting for age and a history of severe chronic health conditions. The UK specific coefficients for APACHE II [18], which have been calibrated for patient admissions in the CMP database, were utilised. Admissions were excluded from the APACHE II model if they were aged under 16 years, involved a CMP unit stay of less than eight hours, or were admissions with burns or following coronary artery bypass grafting. The ICNARC model was developed from the CMP database, and uses measures of acute physiology as well as patient age, diagnostic category, admission source and whether cardiopulmonary resuscitation has been required before admission to calculate a mortality risk for critical care patients. SAPS II is calculated from the patient's age, surgical status, history of chronic disease and 12 acute physiological parameters. The worst values for these parameters in the first 24 hours of admission to the critical care unit are used to calculate the score. Admissions were excluded from the SAPS II model if the patients concerned were aged under 18 years, admitted for coronary care, burns or following cardiac surgery, or were transferred from another hospital. The APACHE II model and ICNARC physiology score estimate ultimate hospital mortality, while the SAPS II model estimates hospital mortality within the same acute hospital and so has a lower observed mortality rate.

#### Outcomes

Mortality data were collected for discharge from critical care and final discharge from the acute hospital. The number of patients receiving mechanical ventilation at any time during the first 24 hours of their CMP unit stay was obtained, as well as the number of these patients receiving both mechanical ventilation and with a partial pressure of arterial oxygen (PaO_2_):fraction of inspired oxygen (FiO_2_) ratio of less than 26.6 kPa, as an indication of how many of these individuals may have developed acute respiratory distress syndrome (ARDS). The length of stay in the CMP unit was calculated in fractions of days from the date and time of admission and discharge. The length of total acute hospital stay was calculated in complete days from the date of admission and final discharge from acute hospital.

Readmissions during the same hospital stay were linked to the index admissions using postcode, date of birth and sex, and confirmed by the appropriate critical care unit. As before, information on patient characteristics and mortality during readmissions was extracted and supplemented with the reasons given for readmission. Readmissions were grouped into primary surgical complications by type or medical complications by system. Where both a surgical and medical complication were listed in the database, the surgical cause was taken as the likely primary reason for readmission.

### Statistical analysis

Statistical analysis was carried out in accordance with an analysis plan agreed by the investigators *a priori*.

#### Descriptive analyses

Summary statistics on patient characteristics and outcomes were presented for all admissions, and stratified by those patients requiring only a single critical care stay, and first and subsequent stays of those patients requiring readmission. Mortality data was also grouped by year of admission. In order to exclude the effect of varying numbers and types of units submitting data on any trends seen over time, a sensitivity analysis was performed using only those units that had submitted data for each year from 1999 to 2005. Prognostic scores were presented as arithmetic means and standard deviations, while lengths of stay were given as medians and interquartile ranges more appropriate to their skewed distribution.

#### Risk factor analysis

Multiple logistic regression analysis was used to assess the relationship between patient characteristics on admission to critical care and mortality at the point of discharge from acute hospital care. The analysis was restricted to the first admission after oesophageal surgery and continuous data were grouped into categories in case the association with mortality was not linear in nature. Categories were determined from plots of the relationship between each continuous variable and outcome to which a flexible smooth function (a generalised additive model with five degrees of freedom) was fitted to the log odds of hospital mortality.

#### Prognostic model evaluation

The performance of the three risk prediction models was compared in this group of patients by measuring each model's discrimination (c statistic [19]), calibration (Hosmer-Lemeshow goodness-of-fit test [20], Cox's calibration regression [21]) and overall goodness-of-fit (Brier's score [22]). The analysis was again restricted to a patient's first admission to critical care following surgery. To facilitate comparison, these analyses were performed using only those admissions meeting criteria for all three models. Subsequently because admissions from the CMP database had been used to derive the ICNARC physiology score, a sensitivity analysis was performed in which the statistical tests were repeated on admissions which had not been used for this purpose.

All analyses were performed using Stata 10.0 (StataCorp LP, College Station, Texas, USA).

## Results

### Patient characteristics

Between 1995 and 2007, 7,227 patients were admitted to 181 critical care units following elective oesophageal surgery for malignancy. This represents 1.28% of the total admissions during this period. Table 1 presents the patient demographic details, severity of illness scores, ventilation status, and survival and length of stay data for primary admissions and readmissions. The mean age of the patients was 64 years and 75% were male. The median length of critical care unit stay for all patients was 2.8 days and the median hospital stay was 17 days.

**Table 1:** Case mix, outcome, treatment and length of stay for patients admitted to critical care following oesophageal surgery for malignancy

		All admissions n = 7227	Single admissions n = 6287	Readmitted patients - first admission n = 873	Readmitted patients - subsequent admissions n = 873
*Case Mix*					
Age (years), mean (SD)		64.3 (9.9)	64.2 (9.9)	65.6 (9.7)	-
Sex (male), n (%)		5429 (75.1)	4752 (75.6)	629 (72.1)	-
APACHE II score, mean (SD)		13.9 (4.8)	13.8 (4.9)	14.3 (4.5)	19.0 (6.4)
ICNARC physiology score, mean (SD)		12.4 (5.6)	12.3 (5.6)	12.8 (5.3)	20.0 (9.3)
SAPS II score, mean (SD)		25.1 (10.5)	25.0 (10.6)	25.8 (10.1)	39.3 (15.6)

*Mortality*					
Unit mortality, n (%)		320 (4.4)	314 (5.0)	-	216 (24.7)
Ultimate acute hospital mortality, n (%)		778 (11.0)	483 (7.8)	289 (33.9)	-

*Treatment*					
Received mechanical ventilation at any time during the first 24 hours of admission, n (%)		3572 (49.4)	3114 (49.5)	424 (48.6)	528 (60.5)
Received mechanical ventilation at any time during the first 24 hours of admission & P/F ratio < 26.6 kPa, n (%)		1470 (20.3)	1253 (19.9)	195 (22.3)	428 (49.0)

*Activity*					
Unit length of stay (days), median (IQR)	Unit survivors	2.8 (1.1 to 5.0)	2.8 (1.1 to 5.0)	2.0 (1.0 to 4.4)	6.0 (2.2 to 12.8)
	
	Unit non-survivors	10.9 (5.1 to 18.9)	10.9 (5.1 to 18.9)	-	6.0 (1.6 to 17.1)
	All	2.8 (1.1 to 5.2)	2.9 (1.1 to 5.5)	2.0 (1.0 to 4.4)	6.0 (2.1 to 13.2)
Total acute hospital length of stay (days), median (IQR)	Hospital survivors	17 (14 to 27)	17 (14 to 27)	38 (23 to 59)	-
	Hospital non-survivors	20 (12 to 37)	16 (9 to 33)	25 (15 to 44)	-
	All	17 (14 to 28)	17 (13 to 24)	33 (20 to 55)	-

Data are presented for all admissions, for those patients that only had a single admission and those that required readmission. APACHE = Acute Physiology And Chronic Health Evaluation; ICNARC = Intensive Care National Audit & Research Centre; IQR = interquartile range; P/F ratio = partial pressure of arterial oxygen (PaO2) divided by the fraction of inspired oxygen (FiO2); SAPS = Simplified Acute Physiology Score; SD = standard deviation.

**Figure 1:**
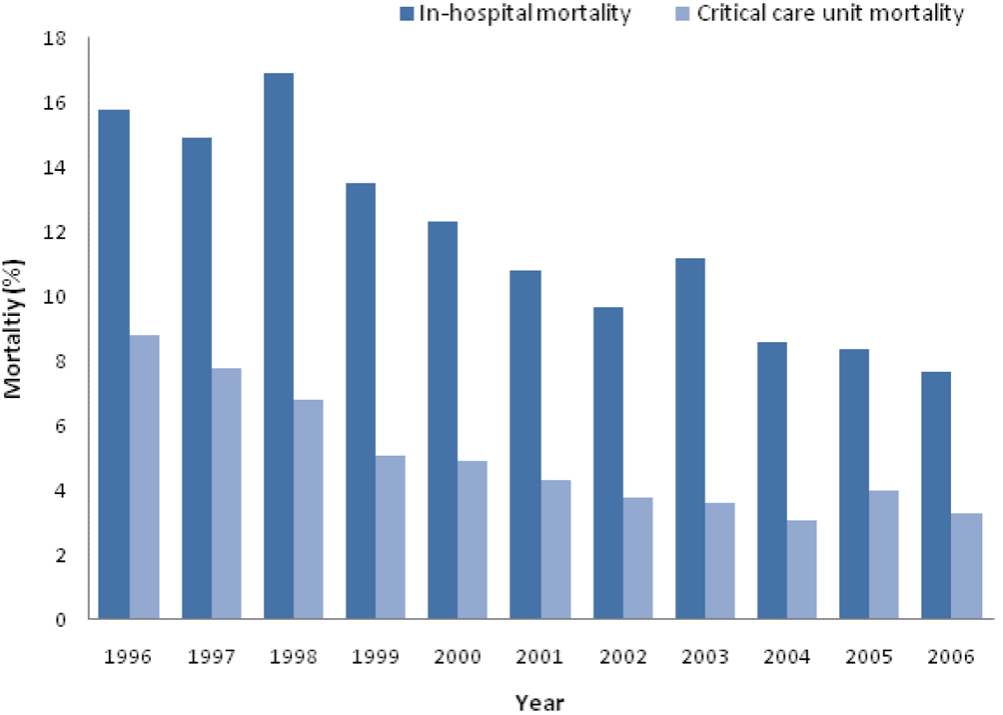
Critical care unit and in-hospital mortality by year of admission.

Over the study period, unit mortality was 4.4% and in-hospital mortality was 11%. Mortality in both critical care and hospital fell steadily over time (Figure 1). Restricting this analysis to only those units providing data for all years showed a similar trend over time.

Eight hundred and seventy-three patients (12.2%) required readmission to critical care. These patients had a longer hospital length of stay and higher in-hospital mortality rate (33.9%) than those only requiring a single admission. This group had substantially worse prognostic scores on readmission; however, they were not significantly different at the start of the index admission (Table 1). Respiratory complications accounted for 48.6% of readmissions, while surgical complications were the primary reason for readmission in 25.4% of cases (Table 2).

**Table 2:** Reasons for readmission to critical care following oesophageal surgery for malignancy

Reason	Number of patients	%
Medical		
Respiratory	424	48.6%
Cardiac	64	7.3%
Sepsis, source unspecified	40	4.6%
Renal	9	1.0%
Neurological	11	1.3%
Other medical complication	20	2.3%
Surgical		
Anastomotic leak	171	19.6%
Chyle leak	14	1.6%
Other surgical cause	37	4.2%
Epidural care	13	1.5%
Unspecified	70	8%

### Risk factors for acute hospital mortality

The results of the multiple logistic regression are presented in Table 3. Associations were found between in-hospital mortality and the following risk factors: the age of the patient, the need for mechanical ventilation within the first 24 hours, PaO_2_:FiO_2 _ratio, lowest pH, lowest serum albumin, and raised serum urea and creatinine. Sex and the various past medical conditions recorded within the CMP database were not associated with in-hospital mortality.

**Table 3:** Factors predicting increased risk of acute hospital mortality

				Adjusted	
				
	Number of admissions	Number of deaths	Percentage of deaths	OR (95% CI)	*P *value
Age (years)					< 0.001
< 50	582	31	5.3	Reference	
50 to 59	1651	125	7.6	1.35 (0.84 to 2.17)	
60 to 69	2608	249	9.6	1.68 (1.07 to 2.63)	
70 to 79	2037	328	16.1	2.64 (1.69 to 4.15)	
80 +	217	45	20.7	3.84 (2.14 to 6.87)	

Sex					0.198
Female	1766	218	12.3	1.15 (0.93 to 1.42)	
Male	5329	560	10.5	Reference	

Condition in past medical history					
Chemotherapy	1672	144	8.6	0.80 (0.64 to 1.02)	0.067
Radiotherapy	182	23	12.6	1.38 (0.81 to 2.36)	0.235
Biopsy proven cirrhosis	5	1	20.0	2.10 (0.17 to 25.45)	0.561
Severe cardiovascular disease	27	3	11.1	0.73 (0.16 to 3.40)	0.691
Severe respiratory disease	40	9	22.5	2.27 (0.87 to 5.90)	0.920
Chronic renal replacement therapy	7	3	42.9	3.62 (0.71 to 18.43)	0.121

Mechanical ventilation					0.025
No	3459	284	8.2	Reference	
Yes	3624	492	13.6	1.24 (1.03 to 1.50)	

P/F ratio (kPa)					< 0.001
< 10	105	27	25.7	3.70 (1.70 to 8.07)	
10 to 19	1034	157	15.2	2.65 (1.49 to 4.69)	
20 to 29	2212	259	11.7	2.04 (1.17 to 3.55)	
30 to 39	1942	201	10.4	1.90 (1.08 to 3.33)	
40 to 49	1107	80	7.2	1.42 (0.77 to 2.57)	
50 to 59	349	18	5.2	Reference	
60+	146	12	8.2	1.90 (0.77 to 4.74)	

Lowest arterial pH					< 0.001
< 7.15	132	40	30.3	1.85 (1.13 to 3.01)	
7.15 to 7.24	555	114	20.5	2.00 (1.49 to 2.68)	
7.25 to 7.29	1114	146	13.1	1.42 (1.11 to 1.84)	
7.30 to 7.34	2838	244	8.6	Reference	
7.35 to 7.39	1568	146	9.3	1.29 (1.01 to 1.66)	
7.40 to 7.44	625	56	9.0	1.05 (0.69 to 1.59)	
7.45 +	66	8	12.1	1.98 (0.72 to 5.48)	

Serum albumin (g l^-1^)					< 0.001
< 15.0	586	123	21.0	2.58 (1.87 to 3.55)	
15.0 to 19.9	1426	192	13.5	1.73 (1.31 to 2.29)	
20.0 to 24.9	1857	155	8.4	1.10 (0.83 to 1.46)	
25.0 to 29.9	1265	86	6.8	Reference	
30.0 to 34.9	342	25	7.3	1.15 (0.71 to 1.87)	
35.0 +	71	8	11.3	2.02 (0.91 to 4.48)	

Serum urea (mmol l^-1^)					0.016
< 6.2	4317	390	9.0	Reference	
6.2 to 7.1	886	90	10.2	0.95 (0.72 to 1.26)	
7.2 to 14.3	1194	177	14.8	1.24 (0.96 to 1.60)	
14.4 +	81	25	30.9	2.49 (1.37 to 4.53)	

Serum creatinine (μmol l^-1^)					0.014
< 50	115	15	13.0	1.25 (0.62 to 2.50)	
50 to 99	5014	456	9.1	Reference	
100 to 149	1571	208	13.2	1.17 (0.93 to 1.48)	
150 +	237	70	29.5	2.01 (1.32 to 3.06)	

Area under the receiver operating characteristic curve = 0.71; 95% CI = 0.69 to 0.73. CI = confidence interval; P/F ratio = partial pressure of arterial oxygen (PaO2) divided by the fraction of inspired oxygen (FiO2); OR = odds ratio.

### Evaluation of prognostic models

**Figure 2:**
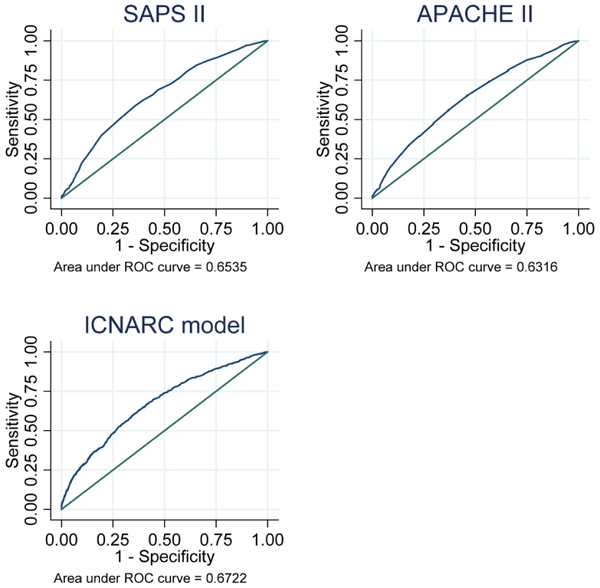
ROC for APACHE II, SAPS II and ICNARC prognostic models. APACHE = Acute Physiology And Chronic Health Evaluation; ICNARC = Intensive Care National Audit & Research Centre; ROC = Receiver operator curves; SAPS = Simplified Acute Physiology Score.

**Figure 3:**
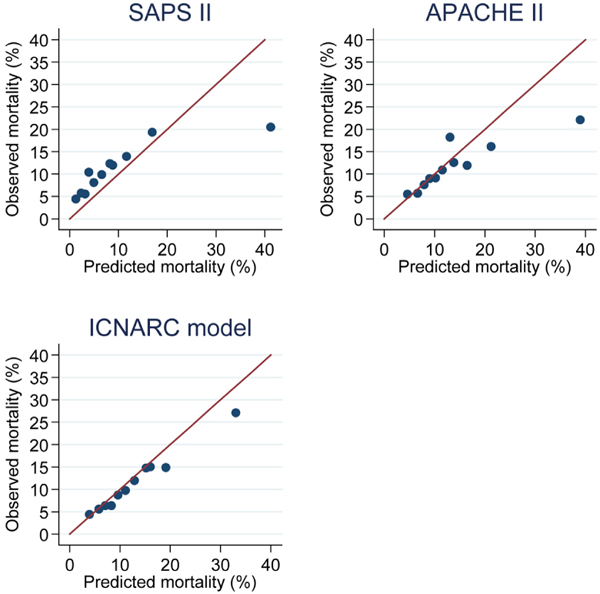
Calibration plots for APACHE II, SAPS II and ICNARC prognostic models. APACHE = Acute Physiology And Chronic Health Evaluation; ICNARC = Intensive Care National Audit & Research Centre; ROC = Receiver operator curves; SAPS = Simplified Acute Physiology Score.

The performance characteristics of the APACHE II, SAPS II and ICNARC models are presented in Table 4. Results are given for the 5,767 admissions eligible for all three of the models. None of the models succeed especially well on tests of either discrimination or calibration; however, the ICNARC physiology score gives the best performance on each of the measures. Receiver operator curves for the models are given in Figure 2 and calibration plots in Figure 3. In the sensitivity analysis carried out on a smaller sample which excluded those admissions used for development of the ICNARC physiology score, all three models performed somewhat less well. However, the order was preserved, with the ICNARC score again giving the best performance on all measures. The areas under the receiver operator curve for the three scores in the sensitivity analysis were ICNARC 0.65 (95% confidence interval (CI) = 0.62 to 0.68), SAPS II 0.63 (95% CI = 0.60 to 0.67) and APACHE II 0.60 (95% CI = 0.57 to 0.63).

**Table 4:** Performance characteristics of APACHE II, SAPS II and ICNARC outcome prediction models

MODEL	APACHE II	ICNARC	SAPS II
Eligible admissions, n (%)	5767 (79.8)	5767 (79.8)	5767 (79.8)
Observed mortality, n (%)	613 (10.6)	613 (10.6)	608 (10.5)
Predicted mortality, n (%)	782.8 (13.6)	729.0 (12.6)	567.0 (9.8)

AUC (95% CI)	0.63 (0.61 to 0.65)	0.67 (0.65 to 0.69)	0.65 (0.63 to 0.67)

Hosmer-Lemeshow C*			
Chi-squared (10)	90.8	29.6	295.7
*P *value	< 0.001	0.001	< 0.001

Cox's calibration regression			
Intercept (95% CI)	-1.01 (-1.22 to -0.80)	-0.42 (-0.64 to -0.20)	-1.12 (-1.30 to -0.95)
Slope (95% CI)	0.59 (0.48 to 0.69)	0.88 (0.77 to 1.00)	0.40 (0.33 to 0.47)
Chi-squared (2)	106.4	27.9	291.4
*P *value	< 0.001	< 0.001	< 0.001

Brier's score	0.097	0.091	0.101

The analysis is performed on those individuals meeting criteria for all three of the models.

APACHE = Acute Physiology And Chronic Health Evaluation; AUC = area under the receiver operating characteristic curve; CI = confidence interval; ICNARC = Intensive Care National Audit & Research Centre; SAPS = Simplified Acute Physiology Score.

## Discussion

This is one of the largest studies to evaluate outcomes following elective oesophageal surgery for malignancy. It is also one of the first to focus on factors in the early postoperative phase and their relationship with subsequent outcomes. From a cohort of 7,227 admissions to critical care between December 1995 and September 2007, we found a critical care mortality rate of 4.4% and hospital mortality rate of 11%. Twelve percent of patients needed at least one readmission to the critical care unit, and these individuals had a much poorer outcome (hospital mortality 33.9%). The median length of critical care unit stay for all patients was 2.8 days, and the median hospital stay was 17 days. Age, evidence of organ dysfunction in the first 24 hours (PaO_2:_FiO_2_, arterial pH, serum urea and creatinine), mechanical ventilation and serum albumin were predictive of an increased risk of death. However, the three prognostic models evaluated were only able to discriminate between patients at low and high risk of mortality to a moderate degree in this group of patients.

The overall in-hospital mortality rate of 11% is broadly in line with recent literature. A worldwide review by Jamieson and colleagues of papers published in the 1990s [4] found an in-hospital mortality of 8.8% (although the authors felt this may have been an underestimate). A large prospective study from the UK (1999 to 2002) recorded an in-hospital mortality rate of 13.7% [23]. Mortality from oesophagectomy has been steadily falling over recent decades [4,24,25], and this trend is also evident across the 11 years of data accessed for the present study. This change is likely to reflect improvements in multi-disciplinary postoperative care, strict patient selection, a degree of selective reporting and the increasing concentration of procedures into high-volume centres, which have repeatedly been shown to have improved outcomes [23,26]. In the UK, centralisation of oesophageal resection was recommended in guidelines published by the NHS Executive in 2001 [27], and subsequently a gradual reorganisation of services has been taking place.

The patients from this study are largely male, as would be expected from the known demographics of oesophageal malignancy. However, the mean age of 64.3 years is lower than the average age among all people with oesophago-gastric cancer in the UK, and is likely to reflect the fact that older patients are more likely to be frail or unfit, and so unable to undergo such a major operative procedure.

The 12% of patients requiring readmission to the critical care unit accounted for almost 40% of all deaths in this cohort. Interestingly, there is little difference in age, sex or prognostic scores between those individuals needing only one admission and those going on to require readmission, suggesting they cannot be distinguished in the immediate postoperative period. The most frequent reasons for readmission in our study were respiratory complications. The respiratory system is well documented in the literature as the most common source of postoperative complication, with an incidence ranging from 12.7 to 40.5% [9,23,28,29]. Work published by Ferguson and Durkin in 2002 quantified the increase in postoperative mortality due to pulmonary complications as a rise from 7% to 32% [28]. Our study emphasises that these complications frequently occur late, after the point at which the patient has been considered well enough for discharge back to the ward.

The aetiology of respiratory dysfunction following oesophageal surgery is multi-factorial. Contributing factors include intraoperative fluid administration, one lung ventilation, micro aspiration, atelectasis and infection [30]. The pathophysiological characteristics of early respiratory dysfunction after oesophagectomy consist of alveolar inflammation, increased alveolar-capillary permeability and impaired alveolar fluid clearance which mirror many of the processes seen during early acute lung injury. Indeed, oesophagectomy has previously been suggested as a suitable model for the study of the condition [31]. It has the distinct advantage that the causative insult is predictably timed, unlike the majority of triggers to lung injury. The prevalence of postoperative respiratory dysfunction varies widely between studies according to definitions used, with figures from some of the larger cohorts ranging between 6.5% and almost 40% [32-35]. In the present study, one-fifth of patients had a severe impairment of oxygenation (PaO_2_:FiO_2 _ratio < 26.6 kPa) and required mechanical ventilation during the first 24 hours of admission. The importance of early respiratory dysfunction is highlighted by the finding that mechanical ventilation and PaO_2_:FiO_2 _ratio in the early postoperative course are independent predictors of outcome. A number of previous studies have suggested preoperative lung function correlates with outcome [28,36,37] and this should be considered when assessing patients for surgery.

There have been relatively few previous multiple logistic regression analyses of prognostic indicators for patients undergoing oesophagectomy reported in the literature. Indicators commonly found to correlate with outcome include age [5,6,9], performance status [5,6,9], tumour stage [5,7,8,23] and a range of comorbidities [7-10]. Consistent with previous work, we found that increasing age was an independent predictor of mortality, although the CMP database does not include the performance status of the patient, which is likely to be correlated with age. Gender was not found to be a significant predictor of outcome, again in agreement with the majority of the literature. There is some evidence to suggest that chemotherapy received in the six months prior to admission to a critical care unit is associated with a reduced risk of hospital mortality for these patients. In more recent years, it has become standard practice to treat patients with oesophageal malignancy with chemotherapy, but during the early years of the CMP database it was not as widely accepted. This change in practice over the time that data have been collected in the CMP database could explain why the observed association is not stronger.

This study differs from previous studies in that it focused on factors in the early postoperative phase. In addition to respiratory dysfunction, pH, renal dysfunction (raised serum urea and creatinine) and low serum albumin were found to be independent predictors of postoperative death. Serum albumin levels fall as part of the acute phase response to inflammation, and its association with mortality may reflect a role as a marker of severity of host response to the surgical insult. In a retrospective analysis of postoperative complications in a cohort of 200 patients undergoing oesophagectomy Ryan and colleagues [35] identified postoperative albumin was similarly associated with increased hospital mortality. By contrast, preoperative albumin levels did not predict either postoperative levels or an increased risk of death. Further work to characterise the relationship between patient factors, operative care, early postoperative organ dysfunction and outcome is required. A better understanding of the inter-relationship between these variables may ultimately improve patient selection or guide treatment.

The mean prognostic scores for first admissions in our patient sample are lower than the average scores for the entire database, as might be expected from a group of elective postoperative patients. Prognostic scores and outcomes for patients undergoing subsequent readmissions are much poorer, and very similar to the averages for the full CMP database [12]. All three of the prognostic models assessed performed poorly on all the tests of discrimination, calibration and goodness of fit. This may reflect the use of these generic models on a less diverse patient population, admitted electively following a major surgical procedure. Alternatively, it may be because important postoperative complications such as pneumonia and anastomotic leak will take time to develop, and will not necessarily be evident on indices measured within 24 hours of surgery. The performance and utility of these scores could be greatly improved by the integration of further preoperative and intraoperative variables. The development of a more accurate model would inform patient management, facilitate audit and improve understanding of the factors that impact outcomes following surgery, which in turn could lead to improvements in practice.

The study has some inherent weaknesses. We have calculated that the CMP database has an average coverage of 45% of general adult critical care units during the period studied, and the submissions studied are therefore representative of approximately 16,000 patients admitted to critical care following oesophagectomy. Although this number includes the majority of operations performed in England, Wales and Northern Ireland, caution should be taken before extrapolating the results of the study to all patients undergoing operations in these countries. Surgical intensive care and high-dependency units do not submit data to the CMP, but receive significant numbers of postoperative oesophagectomy patients. This is particularly common following procedures carried out by thoracic surgeons, and the omission of these cases may bias the wider interpretation of the study.

In addition the CMP database does not code for specific operative procedures and it is possible that the search strategy included individuals who had operative oesophageal procedures in the context of malignancy, other than an oesophagectomy, but were still admitted to a CMP unit. An example might be an admission following a procedure in which resection was not attempted due to tumour progression, but the individual still required postoperative admission to critical care. Consequently, although such cases would be a small proportion of the sample, caution is needed when extrapolating these data to all patients undergoing oesophagectomy.

Furthermore, while the CMP database includes both intensive care and high-dependency beds, it is likely that higher level care is relatively over represented, such that the results may reflect a postoperative population that has a worse than average postoperative course. Finally, it is also important to note that the data were collected over 12 years, and changes in anaesthetic, surgical and general hospital practice over this period may have affected outcomes.

## Conclusions

Mortality among patients admitted postoperatively to critical care in England, Wales and Northern Ireland following elective oesophageal surgery for malignancy between 1995 and 2007 was 11%, although this figure fell steadily over time. One in eight patients required more than one admission. Readmission was most commonly precipitated by respiratory and surgical complications and was associated with a substantial increase in mortality. Patient and perioperative factors predicting an increased risk of death were age, early organ dysfunction, mechanical ventilation and serum albumin level. Established critical care multivariate mortality prediction models performed poorly in this patient cohort.

## Key messages

• Mortality for patients admitted to critical care following oesophagectomy for malignancy between 1995 and 2007 was 11%.

• Readmission was associated with a substantial increase in mortality.

• Factors predicting mortality were age, early organ dysfunction, mechanical ventilation and serum albumin.

• Multivariate mortality prediction models performed poorly in this patient cohort.

## Abbreviations

APACHE II: Acute Physiology and Chronic Health Evaluation II; ARDS: acute respiratory distress syndrome; CI: confidence interval; CMP: Case Mix Programme; FiO_2_: fraction of inspired oxygen; ICNARC: Intensive Care National Audit and Research Centre; PaO_2_: partial pressure of arterial oxygen; SAPS II: Simplified Acute Physiology Score II.

## Competing interests

The authors declare that they have no competing interests.

## Authors' contributions

DP was involved in designing the study and drafted the manuscript. CW and DH were involved in study design and carried out the statistical analysis. TP contributed to the study analysis. DAC helped extensively in drafting the manuscript. DA and FG helped design the study and draft the manuscript. KR helped design the study and coordinated the analysis. GDP devised the study and helped to draft the manuscript. All authors read and approved the final manuscript.
